# The Longitudinal Impact of Parenting Styles on Pathological Internet Use Among College Students: The Mediating Role of Rumination and the Moderating Role of Environmental Sensitivity

**DOI:** 10.3390/bs15111549

**Published:** 2025-11-13

**Authors:** Xiaomin Ke, Zhenhong Wang

**Affiliations:** 1 School of Psychology, Shaanxi Normal University, Xi’an 710062, China; kexiaomin5@hotmail.com; 2 Shaanxi Provincial Key Research Center of Child Mental and Behavioral Health, Xi’an 710062, China; 3 Mental Health Education Center for College Students, Ankang University, Ankang 725000, China

**Keywords:** parenting styles, pathological internet use, rumination, environmental sensitivity, college students

## Abstract

Based on the cognitive-behavioral model of pathological internet use (PIU), this study examined the longitudinal impact of parenting styles on college students’ PIU and the roles played by rumination and environmental sensitivity. A total of 652 freshmen were tracked three times over one year by means of Parenting Style Questionnaire, the Pathological Internet Use Scale, the Rumination Thinking Scale and the High Sensitivity Personality Questionnaire. T1 positive parenting significantly negatively predicted T3 PIU, while T1 negative parenting significantly positively predicted T3 PIU; T2 rumination partially mediated the longitudinal relationship between T1 parenting styles and T3 PIU; environmental sensitivity plays a moderating role in both the direct pathway and the first half pathway of the mediating effect of parental rearing styles on pathological Internet use through rumination thinking, and the results of the moderating effect support the differential susceptibility model. We found that the indirect effect of parenting styles on PIU among college students through rumination was moderated by environmental sensitivity. Specifically, environmental sensitivity strengthened the pathway from parenting styles and rumination to PIU.

## 1. Introduction

Pathological Internet Use (PIU) refers to excessive time spent online by individuals, leading to negative effects on an individual’s physical, psychological, and social functioning (Davis, 2001; Chen, 2006). As of December 2024, China had 1.108 billion internet users, with college students being a major user group (China Internet Network Information Center, 2025). The college years serve as a critical period for forming social connections, and excessive internet use can lead to both internalizing and externalizing problems (Deng et al., 2020; M. Wang et al., 2014). Therefore, it is imperative to explore the mechanisms behind PIU in college students to develop effective prevention and intervention strategies.

Davis (2001) proposed a cognitive-behavioral model of PIU, which distinguishes between specific PIU and generalized PIU. The former refers to dependency on specific online functions and is a kind of special stimulus behavior disorder. The latter takes the form of using the internet as a primary means of connection with the outside world with excessive time consumption. Davis proposed that more attention should be paid to the negative outcomes resulting from generalized PIU (Davis, 2001; Chen, 2006), which aligns with college students’ internet use situation. College students obtain useful information through the internet and engage in interpersonal communication via social software. They are prone to become immersed in establishing and developing interpersonal relationships within social networks, which negatively affects their studies and daily life (Guan, 2021). This phenomenon is more pronounced in the initial stages of college enrollment. Freshmen are in the transitional stage between adolescence and adulthood (emerging adulthood). Due to pressures such as role change, identity formation, and environmental adaptation, they become a susceptible group for pathological internet use (Morgan & Cotton, 2003; Ni et al., 2009). However, excessive internet use by freshmen significantly affects their academic performance and psychological state throughout the entire university stage (L. Zhang et al., 2024). Furthermore, the cognitive–behavioral model proposes that the formation of PIU is a dynamically developing process, in which both distal causes (psychological pathogens and situational cues) and proximal causes (maladaptive cognition) jointly play a role, with maladaptive cognition serving an important function in the formation of PIU. This theory has received widespread attention and support from numerous cross-sectional studies (H. Wei et al., 2022; Mai et al., 2012). However, to further explore the dynamic development process of PIU, conducting only cross-sectional studies is insufficient, and it is necessary to clarify the longitudinal mechanisms of how distal and proximal causes affect PIU. Therefore, this study selects freshmen as the target group and conducts a one-year longitudinal investigation to examine the mechanisms underlying the formation of PIU among college students and to provide support for effective intervention in PIU.

### 1.1. Parenting Styles and College Students’ PIU

The cognitive–behavioral model proposes that parenting style is one of the distal factors influencing the development of an individual’s PIU. Individuals lacking family support are more likely to develop generalized PIU behaviors (Davis, 2001; Chen, 2006). Previous research has shown that adolescents’ perceived intrusive parenting behaviors (e.g., psychological control) are significantly positively correlated with problematic internet use (Cetinkaya, 2019; X. Jiang et al., 2022). Moreover, different parenting styles moderate the amount of time college students spend online (H. Zhang et al., 2023). On the other hand, positive parenting styles negatively predict PIU. Parental warmth is significantly negatively correlated with internet addiction and can significantly negatively predict internet addiction behaviors one year later (Sebre et al., 2023; Lukavská et al., 2022). While these studies reveal a correlation between parenting styles and PIU, few have examined the underlying mechanisms. Therefore, this study uses parenting style as a predictor to investigate its longitudinal impact on PIU.

### 1.2. The Mediating Role of Rumination

Rumination is a typical form of maladaptive cognition, specifically referring to an individual’s persistent and uncontrollable overthinking about the causes, consequences, and implications of negative events they have experienced (Nolen-Hoeksema & Lyubomirsky, 2008). Existing research indicates that parenting style plays an important role in the development of children’s ruminative tendencies. Individuals subjected to excessive parental control are less able to manage negative events later in life, making them more prone to rumination (Nolen-Hoeksema et al., 1995). Excessive parental control and negative emotional expression affect children’s emotion regulation strategies, undermining their confidence in self-regulation abilities and thereby leading to rumination (Hilt et al., 2012).

Rumination has also been proven to predict an individual’s internet use. When experiencing stress or negative emotions, individuals tend to repetitively reflect on the underlying reasons. Individuals share personal information on social media platforms in hopes of receiving feedback, and due to concerns about not receiving positive responses, they frequently check for messages, which increases their usage of social media (Kircaburun et al., 2019; Majid et al., 2020).

However, most previous studies have examined the mediating role of rumination between parenting styles and PIU from a cross-sectional perspective, with greater emphasis on the impact of negative parenting styles on rumination and less attention to the role of positive parenting styles. Therefore, this study employs a longitudinal design and investigates whether rumination mediates the effects of both positive and negative parenting styles on PIU.

### 1.3. The Moderating Role of Environmental Sensitivity

When environmental factors (such as parenting styles) and individual factors (such as maladaptive cognition) interact to influence behavioral development, individual differences can lead to varied outcomes. Environmental sensitivity, defined as an individual’s capacity to perceive and process external environmental stimuli, is considered a key factor contributing to these divergent behavioral outcomes (Pluess et al., 2018). The differential susceptibility model suggests that highly sensitive individuals possess high plasticity. Children who are more vulnerable to adverse effects under negative environmental conditions are also more likely to benefit from supportive environments (Belsky, 1997; Zhou et al., 2016). For example, highly sensitive children exhibit more externalizing problems under negative parenting styles but fewer under positive parenting styles (Slagt et al., 2018). Therefore, in the context of this study, highly sensitive individuals may be more susceptible to the influence of negative parenting styles and more likely to develop PIU; conversely, under positive parenting styles, they may be less prone to PIU.

Pluess proposed the model of environmental sensitivity as a meta-framework to elaborate the specific mechanisms of environmental sensitivity (Z. Wang, 2022). Individuals with high environmental sensitivity are more aware of subtle environmental cues and process environmental information more deeply (Greven et al., 2019). According to the response styles theory of rumination, the more deeply an individual experiences stressful events, the more likely they are to dwell on and repeatedly think about negative emotions (Guo & Wu, 2011). In other words, individuals with higher environmental sensitivity experience parenting practices more intensely. They may be more prone to develop rumination under negative parenting, while positive parenting may help mitigate the formation of rumination. Therefore, this study hypothesizes that environmental sensitivity may moderate the influence of parenting styles on both rumination and PIU.

In summary, based on the propositions of cognitive–behavioral model of PIU and the differential susceptibility model, this study intends to conduct a longitudinal investigation among first-year university students to examine the long-term impact of parenting styles on PIU and to explore the underlying mechanisms involving rumination and environmental sensitivity. Integrating the previous discussions, the following hypotheses are proposed: (1) Parenting styles (T1) longitudinally predict the level of PIU (T3) among university students. Specifically, positive parenting styles negatively predict PIU, while negative parenting styles positively predict PIU. (2) Rumination (T2) mediates the longitudinal relationship between parenting styles (T1) and PIU (T3). More specifically, negative parenting styles increase the level of PIU by exacerbating rumination, whereas positive parenting styles reduce the level of PIU by alleviating rumination. (3) Environmental sensitivity (T1) moderates both the first half of the mediation pathway and the direct path.

## 2. Materials and Methods

### 2.1. Participants

Using a convenience sampling method, a total of 675 freshmen from 10 randomly selected classes at a university in City A, Shaanxi Province, were included for three survey waves. In October 2024 (T1), parenting styles, rumination, highly sensitive personality, and PIU were assessed. Follow-up surveys measuring rumination and PIU were conducted in December 2024 (T2) and June 2025 (T3). Due to reasons such as transferring majors or absence, 23 participants were lost across the three waves. Ultimately, 652 participants (30.2% male, mean age = 18.30 ± 0.68 years) completed all surveys, with an attrition rate of 3.4%.

### 2.2. Measures

#### 2.2.1. Parenting Style Questionnaire

The Chinese version of the Simplified Parenting Style Evaluation Scale was adopted. This scale was developed by Arrindell et al., and the Chinese version was revised by J. Jiang et al. (2010). The scale contains 42 items (21 for father and 21 for mother) across three dimensions: rejection (12 items), emotional warmth (14 items), and overprotection (16 items). In this study, emotional warmth represented positive parenting, while rejection and overprotection represented negative parenting. Each item was rated on a 4-point scale from “never”, “sometimes”, “often” to “always” (scored 1–4). Total scores ranged from 21 to 84, with higher scores indicating higher levels of positive or negative parenting. The internal consistency of the negative and positive parenting dimensions in this study was satisfactory, with Cronbach’s alpha coefficients both being 0.80. The confirmatory factor analysis further indicated good model fit at Time 1 (χ^2^/df = 1.99, *p* < 0.001, CFI = 0.97, TLI = 0.97, RMSEA = 0.040).

#### 2.2.2. Pathological Internet Use Scale (APIUS)

The APIUS, developed by Lei and Yang (2007), was used in this study. It consists of 38 items rated on a 5-point Likert scale from “completely no” to “completely yes” (scored 1–5). Total scores range from 38 to 190. The average score of all items was calculated, with higher scores indicating more severe PIU. The Cronbach’s α values for this scale in the present study were 0.96, 0.94, and 0.95 across the three time points. The confirmatory factor analysis results indicated excellent model fit at all three time points: Time 1: χ^2^/df = 1.95, *p* < 0.001, CFI = 0.97, TLI = 0.96, RMSEA = 0.038; Time 2: χ^2^/df = 1.93, *p* < 0.001, CFI = 0.97, TLI = 0.96, RMSEA = 0.038; Time 3: χ^2^/df = 1.98, *p* < 0.001, CFI = 0.96, TLI = 0.96, RMSEA = 0.039.

#### 2.2.3. Ruminative Response Scale (RRS)

The Chinese version of the RRS, revised by Han and Yang (2009), was employed. This scale has demonstrated good reliability and validity across various studies. It includes 22 items grouped into three factors: obsessive thinking, symptom rumination, and reflective pondering. Responses were made on a 4-point scale, with higher scores indicating higher levels of rumination. The Cronbach’s α values for this scale in the present study were 0.94, 0.92, and 0.94 at T1, T2, and T3, respectively. The confirmatory factor analysis demonstrated adequate model fit at all three assessments: Time 1: χ^2^/df = 2.27, *p* < 0.001, CFI = 0.97, TLI = 0.97, RMSEA = 0.040; Time 2: χ^2^/df = 2.27, *p* < 0.001, CFI = 0.97, TLI = 0.97, RMSEA = 0.038; Time 3: χ^2^/df = 2.28, *p* < 0.001, CFI = 0.97, TLI = 0.97, RMSEA = 0.039.

#### 2.2.4. Highly Sensitive Person Scale (HSP-10)

The HSP-10 is a simplified version of the Highly Sensitive Person Scale (HSPS) developed by Aron and Aron (1997). HSP-10 contains 10 items rated on a 7-point scale from 1 (“very no”) to 7 (“very yes”). The questionnaire comprises two dimensions: ease of excitation (EOE) and aesthetic sensitivity (AES). It is one of the most widely used measures of environmental sensitivity and has shown good validity and reliability. The Cronbach’s α for this scale in the present study was 0.90. The confirmatory factor analysis indicated acceptable model fit at Time 1: χ^2^/df = 2.99, *p* < 0.001, CFI = 0.99, TLI = 0.98, RMSEA = 0.055.

### 2.3. Procedure and Data Analysis

The study was conducted with informed consent obtained from all participants, and an informed consent form was attached to the questionnaire. Each survey was administered by trained experimenters in classroom settings. Questionnaires were distributed and collected on-site, with each session lasting 15–20 min. The same procedure was followed across all three waves. Data were analyzed using SPSS 26.0 and Mplus 8.3.

## 3. Results

### 3.1. Common Method Bias Test

As all data in this study were collected via self-report questionnaires from the same group of participants, common method bias may exist. To mitigate this issue, the experimenters emphasized the confidentiality and anonymity of the surveys during data collection, varied the order of questionnaires across different sessions, and clearly stated that the data would be used solely for scientific research purposes. In data processing, Harman’s single-factor analysis method was adopted to conduct a common method deviation test for the three measurements. The results showed that 17, 9, and 9 factors with eigenvalues greater than 1 were extracted for T1, T2, and T3, respectively. The variance explained by the first factor was 28.34%, 22.49%, and 19.29% for each time point, all below the critical threshold of 40%. These results indicate that no severe common method bias was present in this study.

### 3.2. Descriptive Statistics and Correlation Analysis

Correlation analyses were conducted among the key variables. The means, standard deviations, and correlation matrix for variables across the three time points are presented in Table 1. The results indicated that negative parenting styles, rumination, and PIU were significantly correlated at each time point, which is consistent with the research hypotheses.

### 3.3. Effects of Parenting Styles on College Students’ PIU: Testing the Longitudinal Mediating Role of Rumination

A first-order cross-lagged model was constructed using Mplus 8.3 to test the longitudinal mediation model (see the Supplementary Materials). First, with T1 negative parenting style as the independent variable, the model showed good fit: χ^2^/df = 10.28, CFI = 0.99, TLI = 0.98, SRMR = 0.02, RMSEA = 0.03. After controlling for T1 rumination, T1 negative parenting significantly positively predicted T2 rumination (β = 0.31, *p* < 0.001, 95% CI [0.24, 0.38]). After further controlling for T1 PIU, T2 PIU, and T1 rumination, T2 rumination significantly positively predicted T3 PIU (β = 0.20, *p* < 0.001, 95% CI [0.12, 0.28]), and T1 negative parenting remained a significant positive predictor of T3 PIU (β = 0.17, *p* < 0.001, 95% CI [0.09, 0.25]). These results indicate that rumination partially mediates the relationship between negative parenting and PIU. The longitudinal mediation effect was 0.06, 95% CI [0.03, 0.09].

Next, with T1 positive parenting style as the independent variable, the model also demonstrated good fit: χ^2^/df = 15.29, CFI = 0.99, TLI = 0.98, SRMR = 0.02, RMSEA = 0.05. After controlling for T1 rumination, T1 positive parenting significantly negatively predicted T2 rumination (β = −0.36, *p* < 0.001, 95% CI [−0.43, −0.30]). After further controlling for T1 PIU, T2 PIU, and T1 rumination, T2 rumination significantly positively predicted T3 PIU (β = 0.21, *p* < 0.001, 95% CI [0.13, 0.28]), and T1 positive parenting remained a significant negative predictor of T3 PIU (β = −0.13, *p* < 0.01, 95% CI [−0.21, −0.05]). These results indicate that rumination also partially mediates the relationship between positive parenting and PIU. The longitudinal mediation effect was –0.08, 95% CI [−0.11, −0.04]. Thus, Hypothesis 1 and Hypothesis 2 were supported.

### 3.4. Testing the Moderated Mediation Model

Hierarchical regression analysis was conducted using Mplus 8.3 to examine the moderating effect of T1 environmental sensitivity. First, with T1 negative parenting style as the independent variable (saturated model), the results are presented in Table 2. T1 negative parenting significantly positively predicted T2 rumination (*β* = 0.39, *t* = 11.59, *p* < 0.001), and T2 rumination significantly positively predicted T3 PIU (*β* = 0.28, *t* = 8.13, p < 0.001). The interaction term between T1 negative parenting and T1 environmental sensitivity significantly predicted T2 rumination (*β* = 0.14, *t* = 4.09, *p* < 0.001) and also significantly predicted T3 PIU (*β* = 0.22, *t* = 6.88, *p* < 0.001).

To further explain the substantive nature of the moderating interaction between T1 negative parenting style and environmental sensitivity, values at positive and negative one standard deviation of environmental sensitivity were taken as criteria for high and low environmental sensitivity levels, and simple slope tests were conducted to examine the moderating effect of environmental sensitivity on the relationships between T1 negative parenting style and T2 rumination, as well as between T1 negative parenting style and T3 PIU. The results are presented in Table 3, when environmental sensitivity was at a low level, T1 negative parenting style significantly positively predicted both T2 rumination (β = 0.09, t = 4.16, *p* < 0.001) and T3 PIU (β = 0.13, t = 2.20, *p* < 0.05). When environmental sensitivity was at a high level, T1 negative parenting style continued to significantly positively predict T2 rumination (β = 0.19, t = 6.24, *p* < 0.001) and T3 PIU (β = 0.70, t = 10.70, *p* < 0.001), and these effects were stronger. This indicates that the influence of negative parenting style on both rumination and PIU intensifies as an individual’s level of environmental sensitivity increases.

With T1 positive parenting style as the independent variable (saturated model), the results are presented in Table 4. T1 positive parenting style significantly negatively predicted T2 rumination (β = −0.42, t = −13.21, *p* < 0.001), and T2 rumination significantly positively predicted T3 PIU (β = 0.29, t = 7.90, *p* < 0.01). The interaction term between T1 positive parenting style and T1 environmental sensitivity significantly predicted T2 rumination (β = −0.14, t = −3.92, *p* < 0.001) and also significantly predicted T3 PIU (β = −0.20, t = −6.06, *p* < 0.001).

Values at positive and negative one standard deviation of environmental sensitivity were taken as criteria for high and low environmental sensitivity levels, and simple slope tests were conducted to examine the moderating effect of environmental sensitivity on the relationship between T1 positive parenting style and T3 PIU. The results are presented in Table 5, when environmental sensitivity was at a low level, T1 positive parenting style significantly negatively predicted T2 rumination (β = −0.11, t = −4.58, *p* < 0.05), but its negative predictive effect on T3 PIU was not significant. When environmental sensitivity was at a high level, T1 positive parenting style continued to significantly negatively predict T2 rumination (β = −0.22, t = −6.40, *p* < 0.001) with a stronger effect, and also significantly negatively predicted T3 PIU (β = −0.63, t = −9.25, *p* < 0.001). This indicates that the influence of positive parenting style on rumination intensifies as an individual’s level of environmental sensitivity increases. Hypothesis 3 was supported.

## 4. Discussion

This study found that parenting styles, as a key environmental variable, directly predict college students’ Pathological Internet Use (PIU), which is consistent with previous research findings (Shivam et al., 2021; M. Wei et al., 2017; Y. Li et al., 2014). Positive parenting styles (such as emotional warmth and autonomy support) exert a protective effect against externalizing behavior problems in adolescents by providing emotional resources and psychological support (Hu et al., 2017). In contrast, negative parenting styles (such as psychological control and harsh punishment) positively predict problematic internet use among adolescents (Lo et al., 2021; D. Li et al., 2013). Adolescents under negative parenting conditions are more inclined to use the internet for leisure and exhibit significantly longer usage durations (Feng et al., 2025; Ren & Zhu, 2022). These findings indicate that parenting style is an important predictor of PIU development, and this result provides longitudinal empirical support from a college student population for the cognitive–behavioral model of PIU.

This study also found that rumination plays a partial mediating role in the longitudinal impact of parenting styles on college students’ PIU, indicating that parenting styles influence PIU through rumination. This mechanism aligns with cognitive–behavioral model of PIU. As a maladaptive emotional coping strategy, rumination involves a passive and repetitive focus on the negative emotions triggered by stressful events, rather than taking active steps to alleviate them (Treynor et al., 2003; Ma & Zhou, 2024). Particularly for college students in the transition phase of adapting to university life, negative parenting may contribute to the accumulation of emotional stress, thereby fostering rumination, which in turn can lead to avoidance behaviors such as PIU. In contrast, positive parenting can buffer this pathway. The mediation results are consistent with the theoretical assumptions of the cognitive–behavioral model of PIU. Parenting styles, as a distal factor, not only directly predict students’ PIU levels one year later but also contribute to the development or mitigation of PIU by influencing maladaptive cognition (rumination). This study confirms the critical role of maladaptive cognition in the formation and progression of PIU.

Furthermore, this study found that environmental sensitivity significantly moderates the relationship between parenting styles and college students’ PIU. Simple slope tests indicated that while negative parenting significantly predicted PIU among students with both high and low environmental sensitivity, its effect was stronger for those with high environmental sensitivity. In contrast, positive parenting did not have a significant impact on PIU among students with low environmental sensitivity but showed a significant effect among highly sensitive individuals. Additionally, environmental sensitivity moderated the mediating pathway through which parenting styles influence PIU via rumination. Simple slope tests revealed that for highly sensitive individuals, the positive predictive effect of negative parenting on rumination, as well as the negative predictive effect of positive parenting on rumination, were both strengthened.

The moderating effects observed in this study support the differential susceptibility model, indicating that highly sensitive individuals exhibit heightened responsiveness to environmental influences in both positive and negative directions. They not only show stronger negative reactions to adverse parenting environments, making them more vulnerable to developmental issues such as PIU, but also benefit more significantly from positive parenting contexts (Dong et al., 2024). From a neurophysiological perspective, individuals with high environmental sensitivity possess neurobiological systems that process environmental cues more deeply, and their stress response systems such as the HPA axis and sympathetic nervous system exhibit higher reactivity (Z. Wang et al., 2020). Therefore, when exposed to negative parenting behaviors such as rejection, indifference, or overcontrol, highly sensitive individuals experience more intense negative emotions (e.g., anxiety and depression) and more persistent rumination. This deep processing of negative cognitive–emotional experiences increases their tendency to use the internet as an escape from real-life stress and distress, thereby significantly elevating the risk of PIU (Gao & Chen, 2006). Conversely, in supportive environments characterized by warmth and responsiveness, highly sensitive individuals are more attuned to positive parental signals, which enhances their emotional security and positive self-perception, facilitates the development of adaptive emotion regulation strategies (e.g., cognitive reappraisal), and effectively reduces rumination (J. B. Li et al., 2019). These psychological resources diminish the need to escape or alleviate negative emotions through excessive internet use, thereby more effectively mitigating the occurrence of PIU. From a cognitive–behavioral perspective, highly sensitive individuals demonstrate deep cognitive processing of environmental information, including parental behaviors. In negative parenting contexts, they are more likely to attend to and dwell on negative cues, engage in excessive reflection and catastrophic interpretations, thereby forming and maintaining maladaptive cognitions (e.g., negative self-schemas) and strong ruminative tendencies. In positive parenting environments, however, they process and internalize supportive signals such as warmth and encouragement more profoundly and enduringly. This deep processing of positive information helps construct adaptive cognitive schemas, buffers negative emotions, suppresses rumination, and promotes healthier behavioral choices. Cognitive processes such as rumination often serve as precursors to behavioral outcomes like PIU (Garnefski et al., 2001). The moderated mediation results highlight how environmental sensitivity shapes individual differences in cognitive and behavioral responses, thereby deepening our understanding of the role of individual susceptibility in the development and prevention of PIU.

## 5. Conclusions

However, this study has several limitations. First, all data were collected from self-reports of college students, and the findings may be influenced by expectancy effects. Future research could obtain more objective data by incorporating assessments from multiple informants, such as the individuals themselves, peers, parents, and teachers. Second, the study only recruited first-year students from a single university, which limits the representativeness of the sample and the generalizability of the results to other populations. Future studies should employ large-sample longitudinal designs and include more diverse types of participants. Finally, aside from parental parenting styles, rumination, and environmental sensitivity, other variables—such as psychological vulnerabilities (e.g., negative emotions) and social support—may also influence the formation and development of PIU. Future research should explore the mechanisms of additional factors to provide more perspectives on the prevention and intervention of PIU among university students.

The findings of this study offer several implications for the prevention and intervention of PIU: First, in terms of family education, parents should strive to create a warm and supportive psychological environment at home while minimizing negative parenting behaviors such as rejection and excessive control. This approach can more effectively prevent the development of PIU in adolescents. Second, universities should pay greater attention to mental health education during freshmen’s transition period. Helping new students, especially those experiencing adjustment difficulties, to develop healthy internet usage habits and cognitive–emotional regulation strategies can facilitate a smoother adaptation to university life and reduce the risk of turning to PIU as a maladaptive coping mechanism. Third, when designing interventions for PIU among college students, special attention should be given to individuals with high environmental sensitivity. Interventions could focus on cognitive training approaches, such as mindfulness therapy. For example, mindfulness practices can disrupt automated cognitive processes through four specific mechanisms (Kang et al., 2013), reducing excessive focus on negative emotions and thereby alleviating PIU.

## Figures and Tables

**Table 1:** Means, Standard Deviations, and Correlations for Variables Across Three Time Points.

Variable	M	SD	1	2	3	4	5	6	7	8
1. Negative Parenting (T1)	2.89	0.58	1							
2. Positive Parenting (T1)	2.84	0.54	−0.57 **	1						
3. Environ. Sensitivity (T1)	4.27	1.56	0.12 **	−0.09 *	1					
4. Rumination (T1)	2.86	0.69	0.31 **	−0.32 **	0.06	1				
5. Rumination (T2)	2.78	0.70	0.38 **	−0.43 **	0.09 *	0.33 **	1			
6. Rumination (T3)	2.83	0.70	0.35 **	−0.30 **	0.03	0.14 **	0.40 **	1		
7.PIUT1	3.64	0.73	0.35 **	−0.33 **	0.04	0.34 **	0.22 **	0.23 **	1	
8.PIUT2	3.58	0.74	0.62 **	−0.63 **	0.13 **	0.42 **	0.60 **	0.35 **	0.44 **	1
9.PIUT3	3.61	0.74	0.41 **	−0.40 **	0.01	0.21 **	0.43 **	0.54 **	0.0.23 **	0.50 **

Note: * *p* < 0.05, ** *p* < 0.01.

**Table 2:** Moderated Mediation Effects (Independent Variable: Negative Parenting Style).

	(Equation (1)) Criterion: Rumination T2			(Equation (2)) Criterion: PIU T3		
Predictors	*β*	*t*	95%CI	*β*	*t*	95%CI
Environ. Sensitivity T1	0.05	1.45	[−0.02, 0.1]	−0.04	−1.21	[−0.11, 0.02]
Negative Parenting T1	0.39	11.59 ***	[0.32, 0.45]	0.33	9.50 ***	[0.26, 0.39]
Environ. Sensitivity T1 × Negative Parenting T1	0.14	4.09 ***	[0.08, 0.21]	0.22	6.88 ***	[0.16, 0.29]
Rumination T2				0.28	8.13 ***	[0.21, 0.35]
*R* ^2^	0.17			0.30		

Note: *** *p* < 0.001.

**Table 3:** Direct and Indirect Effects at Different Levels of Environmental Sensitivity (Independent Variable: Negative Parenting Style).

Effect Type	Level of Environmental Sensitivity	β	*t*	95% CI
Indirect Effect	Low (M − 1SD)	0.09	4.16 ***	[0.05, 0.13]
	Mean (M)	0.14	6.37 ***	[0.10, 0.18]
	High (M + 1SD)	0.19	6.24 ***	[0.13, 0.25]
Direct Effect	Low (M − 1SD)	0.13	2.20 *	[0.01, 0.24]
	Mean (M)	0.41	9.16 ***	[0.33, 0.50]
	High (M + 1SD)	0.70	10.70 ***	[0.57, 0.83]

Note: * *p* < 0.05, *** *p* < 0.001.

**Table 4:** Moderated Mediation Effects (Independent Variable: Positive Parenting Style).

	(Equation (1)) Criterion: Rumination T2			(Equation (2)) Criterion: PIU T3		
Predictors	*β*	*t*	95% CI	*β*	*t*	95% CI
Environ. Sensitivity T1	0.06	1.67	[−0.01, 0.13]	−0.03	−0.98	[−0.10, 0.03]
Positive Parenting T1	−0.42	−13.21 ***	[−0.48, −0.38]	−0.26	−7.36 ***	[−0.30, −0.17]
Environ. Sensitivity T1 × Positive Parenting T1	−0.14	−3.92 ***	[−0.20, −0.07]	−0.20	−6.06 ***	[−0.27, −0.14]
Rumination T2				0.29	7.90 ***	[0.22, 0.36]
*R* ^2^	0.21			0.28		

Note: *** *p* < 0.001.

**Table 5:** Direct and Indirect Effects at Different Levels of Environmental Sensitivity (Independent Variable: Positive Parenting Style).

Effect Type	Level of Environmental Sensitivity	*β*	*t*	95% CI
Indirect Effect	Low (M − 1SD)	−0.11	−4.58 ***	[−0.16, −0.07]
	Mean (M)	−0.16	−6.47 ***	[−0.21, −0.12]
	High (M + 1SD)	−0.22	−6.40 ***	[−0.28, −0.15]
Direct Effect	Low (M − 1SD)	−0.09	−1.39	[−0.22, 0.04]
	Mean (M)	−0.36	−7.18 ***	[−0.46, −0.26]
	High (M + 1SD)	−0.63	−9.25 ***	[−0.76, −0.50]

Note: *** *p* < 0.001.

## Data Availability

The data that support the findings of this study are available from the corresponding author upon reasonable request.

## Supplementary Materials

The following supporting information can be downloaded at: https://www.mdpi.com/article/10.3390/bs15111549/s1, Figure S1. Cross-Lagged Model of the Effect of Negative Parenting on College Students’ PIU (***, *p* < 0.001); Figure S2. Cross-Lagged Model of the Effect of Positive Parenting on College Students’ PIU (***, *p* < 0.001).
