# Supplementary material for: The Longitudinal Impact of Parenting Styles on Pathological Internet Use Among College Students: The Mediating Role of Rumination and the Moderating Role of Environmental Sensitivity
Source: Behav Sci (Basel). 2025 Nov 13;15(11):1549. doi: 10.3390/bs15111549 (PMC12649548; doi:10.3390/bs15111549)
Supplement: Supplementary file 1 [file behavsci-15-01549-s001.zip › behavsci-3923764-supplementary.pdf]

Effects of Parenting Styles on College Students' PIU: Testing the Longitudinal Mediating Role of Rumination

A first-order cross-lagged model was constructed using Mplus 8.3 to test the longitudinal mediation model. First, with T1 negative parenting style as the independent variable, the model showed good fit: $\chi^2/df = 10.28$, CFI = 0.99, TLI = 0.98, SRMR = 0.02, RMSEA = 0.03. After controlling for T1 rumination, T1 negative parenting significantly positively predicted T2 rumination ($\beta = 0.31$, $p < .001$, 95% CI [0.24, 0.38]). After further controlling for T1 PIU, T2 PIU, and T1 rumination, T2 rumination significantly positively predicted T3 PIU ($\beta = 0.20$, $p < .001$, 95% CI [0.12, 0.28]), and T1 negative parenting remained a significant positive predictor of T3 PIU ($\beta = 0.17$, $p < .001$, 95% CI [0.09, 0.25]). These results indicate that rumination partially mediates the relationship between negative parenting and PIU. The longitudinal mediation effect was 0.06, 95% CI [0.03, 0.09].

Next, with T1 positive parenting style as the independent variable, the model also demonstrated good fit: $\chi^2/df = 15.29$, CFI = 0.99, TLI = 0.98, SRMR = 0.02, RMSEA = 0.05. After controlling for T1 rumination, T1 positive parenting significantly negatively predicted T2 rumination ($\beta = -0.36$, $p < .001$, 95% CI [−0.43, −0.30]). After further controlling for T1 PIU, T2 PIU, and T1 rumination, T2 rumination significantly positively predicted T3 PIU ($\beta = 0.21$, $p < .001$, 95% CI [0.13, 0.28]), and T1 positive parenting remained a significant negative predictor of T3 PIU ($\beta = -0.13$, $p < .01$, 95% CI [−0.21, −0.05]). These results indicate that rumination also partially mediates the relationship between positive parenting and PIU. The longitudinal mediation effect was −0.08, 95% CI [−0.11, −0.04]. Thus, Hypothesis 1 and Hypothesis 2 were supported.

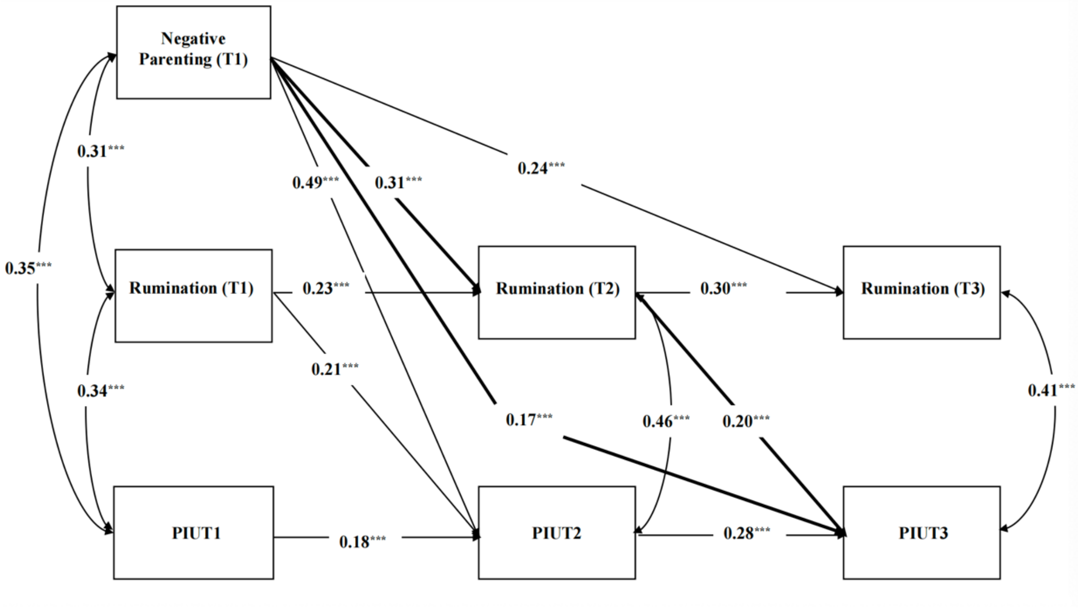

Figure S1: Cross-Lagged Model of the Effect of Negative Parenting on College Students' PIU

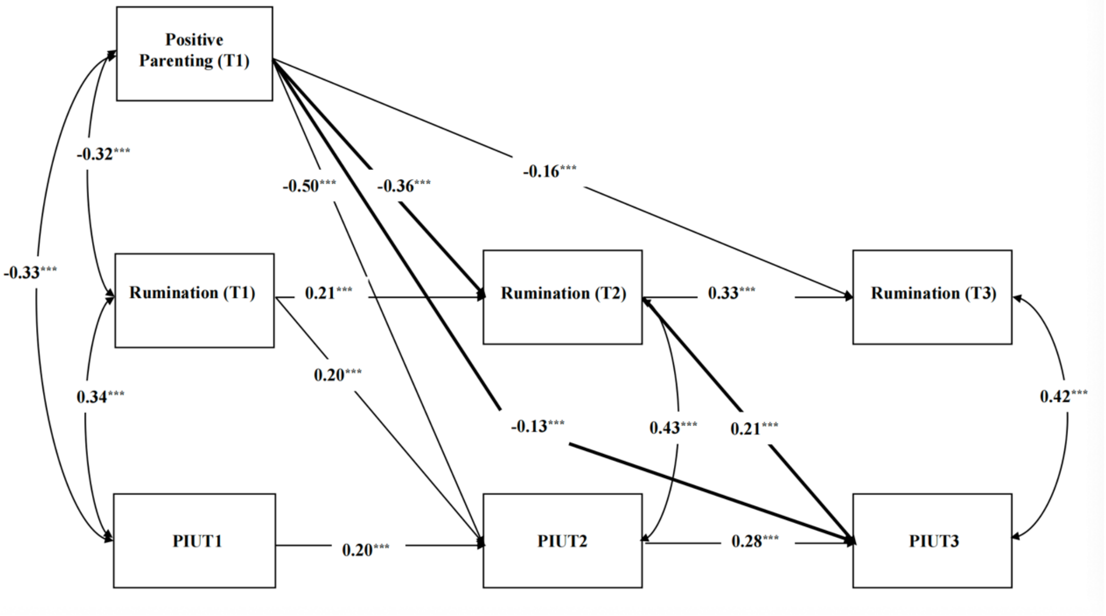

Figure S2: Cross-Lagged Model of the Effect of Positive Parenting on College Students' PIU
